# The Prince of Wales's Hospital Fund for London

**Published:** 1899-10-21

**Authors:** 


### THE PRINCE OF WALES'S HOSPITAL FUND FOR LONDON.

The Hon. Secretaries of the above Fund write: In the report of a conference recently convened of the Hospital Reform Association, the chairman, Dr. W. Knowsley Sibley, is reported to have said, "He was sorry that the managers of the Prince of Wales's Fund were rather adverse from inquiry, maintaining that their functions were purely administrative. He believed the Fund would become much more popular if its managers followed the example of the managers of the Hospital Sunday Fund and gave preference to the hospitals who adopted the inquiry system." We should be glad if you would kindly permit us to correct this. The visitors of each hospital specially report not only whether any attempt is made to ascertain the social circumstances of the in-patients, but also whether there is an almoner or inquiry officer for the out-patients. The Distribution Committee carefully consider the reports of the visitors and allocate their awards after reviewing all matters connected with the hospital, including the question of inquiry.

